# Distal Humerus Morphological Analysis of Chinese Individuals: A Statistical Shape Modeling Approach

**DOI:** 10.1111/os.13492

**Published:** 2022-09-14

**Authors:** Wei Zhao, Yao Guo, Chuangye Xu, Guoxian Pei, Shiva Basnet, Yanjun Pei, Xiuyun Su

**Affiliations:** ^1^ School of Medicine Southern University of Science and Technology Shenzhen China; ^2^ Intelligent and Digital Surgery Innovation Center Southern University of Science and Technology Hospital Shenzhen China

**Keywords:** Digital orthopedics, Elbow hemiarthroplasty, Implant design, Morphology, Statistical shape modeling

## Abstract

**Objective:**

A detailed analysis of the morphology of distal humeral articulation can help in the creation of anatomic prostheses of hemiarthroplasty. This study used statistical shape modeling to evaluate the 3D morphology of the distal humerus in healthy Chinese individuals and to investigate the proper articular morphology differences.

**Methods:**

A statistical shape model (SSM) of the distal humerus was created using CT scans of 106 survey‐confirmed nonpathologic elbows. In addition, the articular components of each principal component (PC) were selected and fitted on the mean mode. The Euclidean point‐to‐mesh distance of articular modes was calculated as a measurement the proper change in the morphology of the articulation.

**Results:**

The first seven PCs jointly accounted for 80.9% of the total variation (44.4%, 12.2%, 7.9%, 5.9%, 4.1%, 3.4% and 3%, respectively). In the mean model, the distance between the medial and lateral epicondyles was 57.4 mm, the width of the articulation was 42.1 mm, and the angle of the transepicondylar line (TEL) and C line was 4.8°. The articular surface differences of the first PC were significant (RMS: 1.43 mm in the −3 SD model and 2.38 mm in the +3 SD model), whereas under other conditions, the differences were not remarkable despite the maximum deformation not exceeding 1 mm.

**Conclusion:**

A novel method (SSM) was used to evaluate the 3D morphology of the distal humerus in healthy Chinese individuals and investigate the proper articular shape differences. We found the proper shape of articular surface basically transformed into one variation pattern which was relevant to the bone size, even though the morphology of distal humerus possessed complicated variation modes. The findings of this study can be helpful to design the next generation of elbow hemiarthroplasty in the future.

## Graphical abstract

Through a statistical shape model (SSM) we found although the distal humeral morphology possessed complex variation modes, the proper shape of the articular surface primarily transformed into one variation pattern. The database we used reflects the population‐specific characteristics of the bony structures, which can be useful in the development of distal humeral hemiarthroplasty implants.

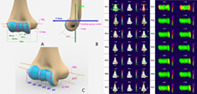

## Introduction

Severe distal humerus fractures are often challenging to treat. In unreconstructible fractures, elbow hemiarthroplasty (EHA) could be a rational option since it can replace the damaged articular surface and have little impact on the contralateral joint.^1^, ^2^, ^3^, ^4^, ^5^ However, total elbow arthroplasty (TEA) is considered a more recommended treatment option,^6^, ^7^, ^8^ even though TEA has a high rate of complications, including aseptic loosening and periprosthetic fracture,^9^ and could lead to lifelong limitations on weight‐bearing activities, which means that the utility of this procedure is only recommended for older patients with sedentary lifestyles.^1^ The main factor restricting the application of EHA is thought to be the inability of current prosthesis of EHA to replicate the native joint mechanics.^10^, ^11^, ^12^

The most common complication of EHA is reported to be cartilage wear on the proximal ulna and radius.^2^ The geometry of current prostheses deviates from the naive anatomy of distal humerus articulation. The prosthesis could induce abnormal contact stress on the contralateral articular surface, thus leading to the destruction of cartilage.^13^, ^14^ Such a situation could be even more serious for Asian patients. Due to differences in bone size and shape,^15^ commercial EHA prostheses based on the Western population are less conforming. This is one of the reasons why EHA cannot be used in China. To address this issue, the morphology of the distal humerus should be accurately quantified based on Chinese‐specific statistical data.

The current literature on anthropometric information of the distal humerus is limited. Wevers *et al*.^16^ and Shiba *et al*.^17^ thoroughly quantified the geometry of elbow articulating surfaces and the anatomy of the ulnohumeral joint on cadaveric humeri. Sabo *et al*.^18^ and Desai *et al*.^14^ performed morphologic analysis of the capitellum and the distal humerus, respectively, using 3‐dimensional imaging techniques. However, currently available studies have focused on discrete parameters (lengths, radii, angles). A statistical shape model (SSM) is a model that represents the mean shape of a population and its modes of variation. It can systematically describe the statistical variations of the anatomic shape and identify relationships between various anatomic features. SSMs have been used to describe shape variation in the bones of the upper extremities.^19^, ^20^, ^21^, ^22^ To our knowledge, however, no studies have used SSMs to analyze 3D morphology and the shape variations of the distal humerus. Therefore, the aims of this study were: (i) to develop SSMs of the distal humerus based on Chinese data to systematically explore the major variations in this bone; (ii) to evaluate the 3D morphology of the distal humerus in healthy elbows; and (iii) to further investigate the proper morphology differences among the articular components by measuring differences in the surfaces in each shape variation.

## Materials and Methods

### Data

This medical imaging study was approved by the Ethics Committee of the Southern University of Science and Technology Hospital (No. of IRB: ECSUSTH‐2022‐064). A waiver of patients' informed consent was granted due to the retrospective study design. The patients' data were anonymized.

A database of healthy distal humeri was established. The inclusion criteria included: (i) 18–60 years old; (ii) no elbow trauma and surgery history; and (iii) no elbow deformity and other pathological changes. One hundred six computed tomography (CT) scans of patients were consisted (72 men and 34 women, 54 left and 52 right, aged between 18 and 56 years, with an average of 33.5 years). All CT scans were performed using a 128‐slice clinical scanner (GE optima 660, New Berlin, WI, USA) at a slice thickness of 0.625 mm. Three‐dimensional surface models of each distal humerus were created using Mimics 24 software (Mimics Innovation Suite; Materialise, Leuven, Belgium) and exported as stereolithography (STL) files. To prepare the development of the SSMs, the right‐side humeri data were reflected to be left‐sided. All models were clipped from the axial plane 50 mm proximal to the vertex of the olecranon fossa to focus on the variations in the distal humerus.

### Statistical Shape Modeling

**Fig. 1:**
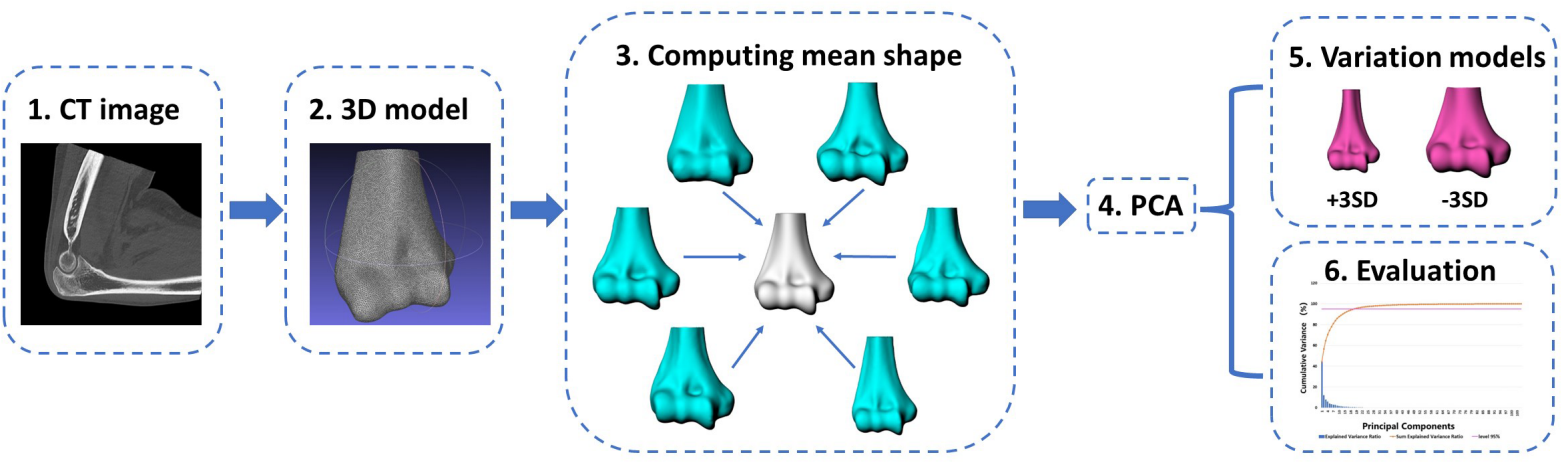
Summarizing a statistical shape analysis framework (SSM). Starting from (1) segmentation of CT images, (2) 3D models' construction and these two steps were repeated for all the patients in the database, generating the inputs for (3) computing the mean shape; (4) PCA involved processes to (5) compare variations in shape (eg, ±3 SD from the mean shape) and (6) perform quantitative assessments.

A baseline 3D surface model was randomly chosen from the database. To build a smooth and uniform topology, the baseline model was remeshed in Geomagic Wrap 2021 (3D system, Rock Hill, SC, USA) with a mean edge length of 0.6 mm, resulting in surface meshes with 22,788 vertexes and 45,358 facets. To create an SSM, a one‐on‐one mesh correspondence between all models was built. First, the other models were registered to the baseline model using Geomagic Wrap 2021. Subsequently, the baseline mesh was morphed to each mesh using R3DS Wrap 3.3 (R3DS, Voronezh, Russia). This process resulted in the meshes for each model having an identical topology but distinctive shapes customized to the original morphology. Finally, the corresponding meshes were imported into SlicerSALT.^23^ The mean shape of each surface model was calculated, and principal component analysis (PCA) was performed to compute the modes of variation which is defined as principal components (PCs) of the shape. For the description of the distal humeral shape, we chose to report in descending order the PCs that represented more than 3% of the total variation. Three distal humerus models were generated for each PC, including the mean shape and ± 3 standard deviations (SD) from the mean, which represented the 99% confidence interval (CI) (Fig. 1).

### Quantitative analysis

The quality of the SSM built for the distal humerus was quantitatively evaluated by three metrics (compactness, specificity and generalization). A compact SSM should have little variance and accurately reconstruct a new shape instance with few shape parameters.^24^ Thus, compactness is defined as a measure of an SSM's efficiency. The generalization quantifies the ability of the SSM to represent a new instance with the same structure.^24^ It is evaluated by performing leave‐one‐out tests on the training data. The specificity measures the validity of the shape instances generated by the SSM.^24^ It is measured by generating a large set of shape instances with the SSM and calculating the average distance between them and their most similar sample in the training data.

### Measurements

**Fig. 2:**
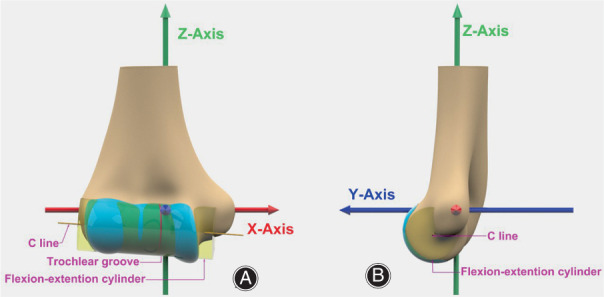
The coordinate system of the distal humerus was created. (A) the anterior–posterior view; (B) the lateral view.

**Fig. 3:**
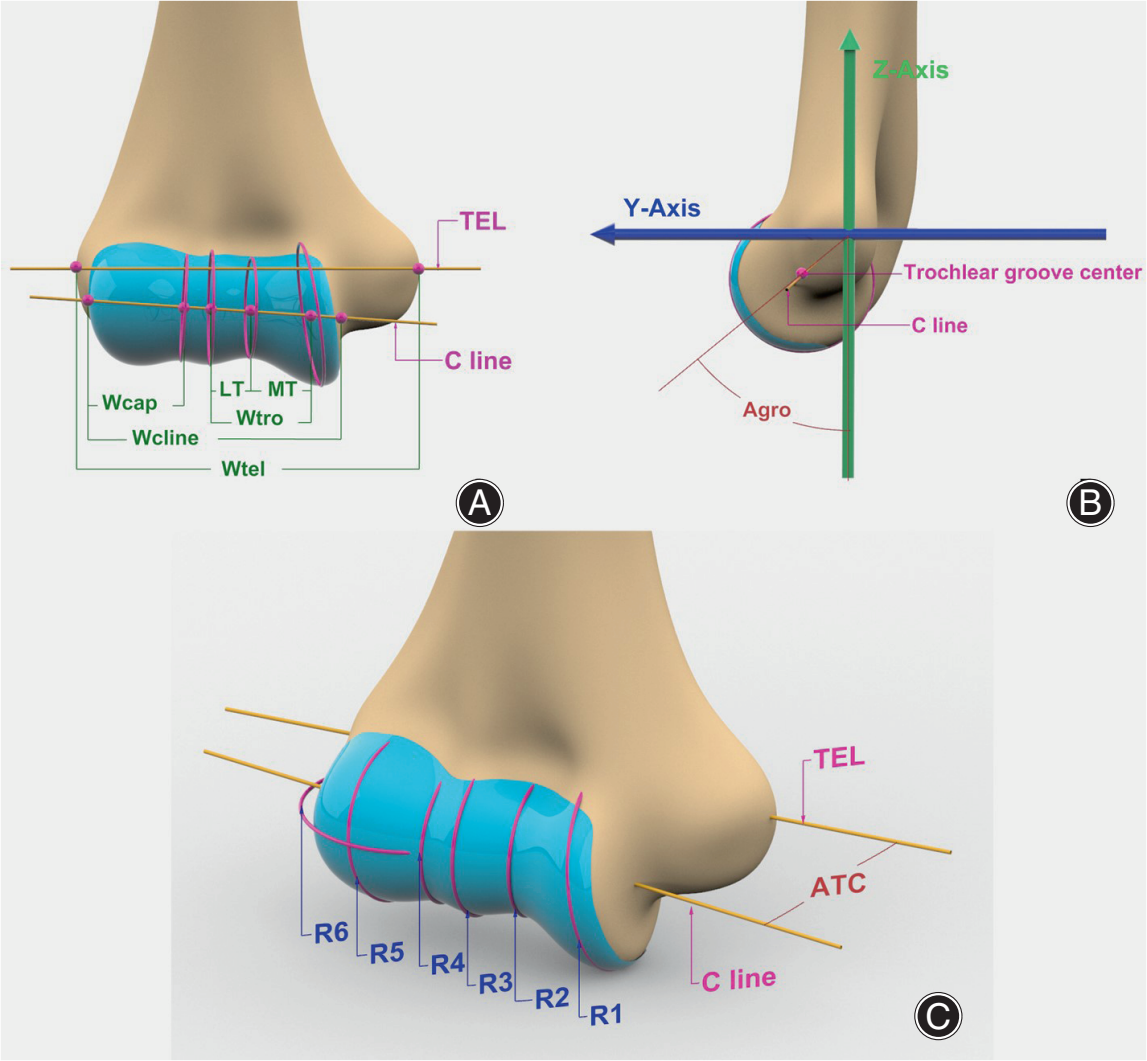
Parameters measured on the distal humerus. (A) the anterior‐posterior view; (B) the lateral view; (C) shows the oblique view.

Measurements were performed on each of the distal humerus models. First, the characteristic points, including the most prominent point of the medial and lateral epicondyles and the centers of the capitellum and trochlear groove, were determined on the model using the semiautomated function of 3‐Matic 16 (Mimics Innovation Suite; Materialise, Leuven, Belgium). The flexion‐extension axis was determined by a cylinder‐fit axis through the articular surface, which was equivalent to the central axis line (C line) of the joint reported by Shiba *et al*.^17^ Second, a coordinate system of the distal humerus was created to provide a measurement reference frame (Fig. 2A,B). The x‐axis was defined as a line joining the most prominent point of the medial and lateral epicondyles (the transepicondylar line, TEL). The y‐axis was set in the sagittal plane passing the trochlear groove center and along the direction of the distal humerus central axis. The z‐axis was perpendicular to the x–y plane, pointing anteriorly (Fig. 2A,B). Consequently, the following parameters were measured in 3‐Matic (Fig. 3A–C):

- Wtel: the distance between the most prominent point of the medial and lateral epicondyles;
- Wcline: the distance along the C line from the medial edge of the trochlea to the lateral edge of the capitellum;
- Wtro: the distance along the C line from the medial trochlear ridge to the lateral trochlear ridge;
- Wcap: the distance along the C line from the groove between the trochlea and capitellum to the lateral edge of the capitellum;
- RT: the ratio between the width of the medial trochlea (MT) and the width of the lateral trochlea (LT);
- RTC: the ratio of Wtro to Wcap;
- ATC: the acute angle of the TEL and C line;
- Agro: the acute angle of the line from the original point to the trochlear groove center and y‐axis;
- R1–R5: the radii of the joint rotation axis at each of five regions, measured by circle fit at the medial trochlear ridge, trochlear groove, lateral trochlear ridge, groove between the trochlea and capitellum and largest radius of the capitellum on the sagittal plane; and
- R6: the largest radius of the capitellum on the transverse plane;

### Statistical Analysis

Two investigators (WZ and YG) selected the points and built three separate coordinate systems on 10 models to assess the intraobserver and interobserver reliability. The intraclass correlation coefficient (ICC) was analyzed using SPSS software (version 24.0, SPSS Inc., Chicago, IL, USA).

### Articular Surface Differences Analysis

**Fig. 4:**
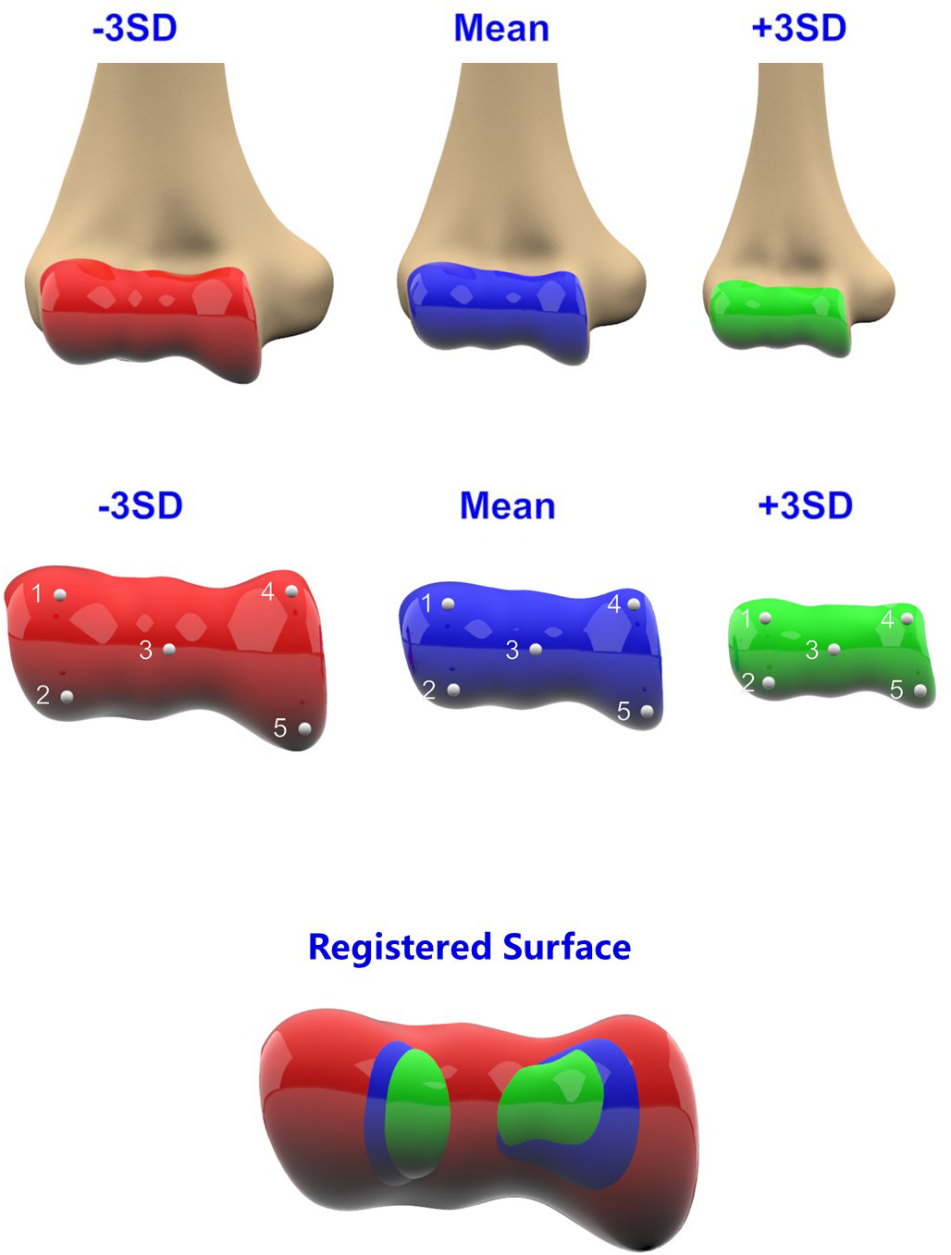
(A) the articular components were selected on the modes; (B) five anatomical landmarks were used to register articular surface; (C) the registered articular surface.

The position and orientation differences among the articular components should be eliminated in each PC to analyze the proper shape difference. Therefore, the articular components (including the trochlea and capitellum) of each PC were selected and fitted on the mean mode before the measurement. The articular surfaces of the mean mode and ± 3 SD modes of each variation were delineated using 3‐Matic. The obtained surface modes were registered using five anatomical landmarks. Figure 4 shows the ones that were used. The Euclidean point‐to‐mesh distance was used from all nodes of the mean articular mode to the ± 3 SD articular surfaces of each variation as a measurement the proper change in the morphology of the articular surface.

## Result

### Consistency Test

Intraobserver reliability for the manual steps of coordinate system creation had an ICC of 0.8 (95% confidence interval, 0.7–1.0), while the ICC of interobserver reliability was 0.9 (95% confidence interval, 0.8–1.0).

### Evaluation of the Statistical Shape Model

**Fig. 5:**
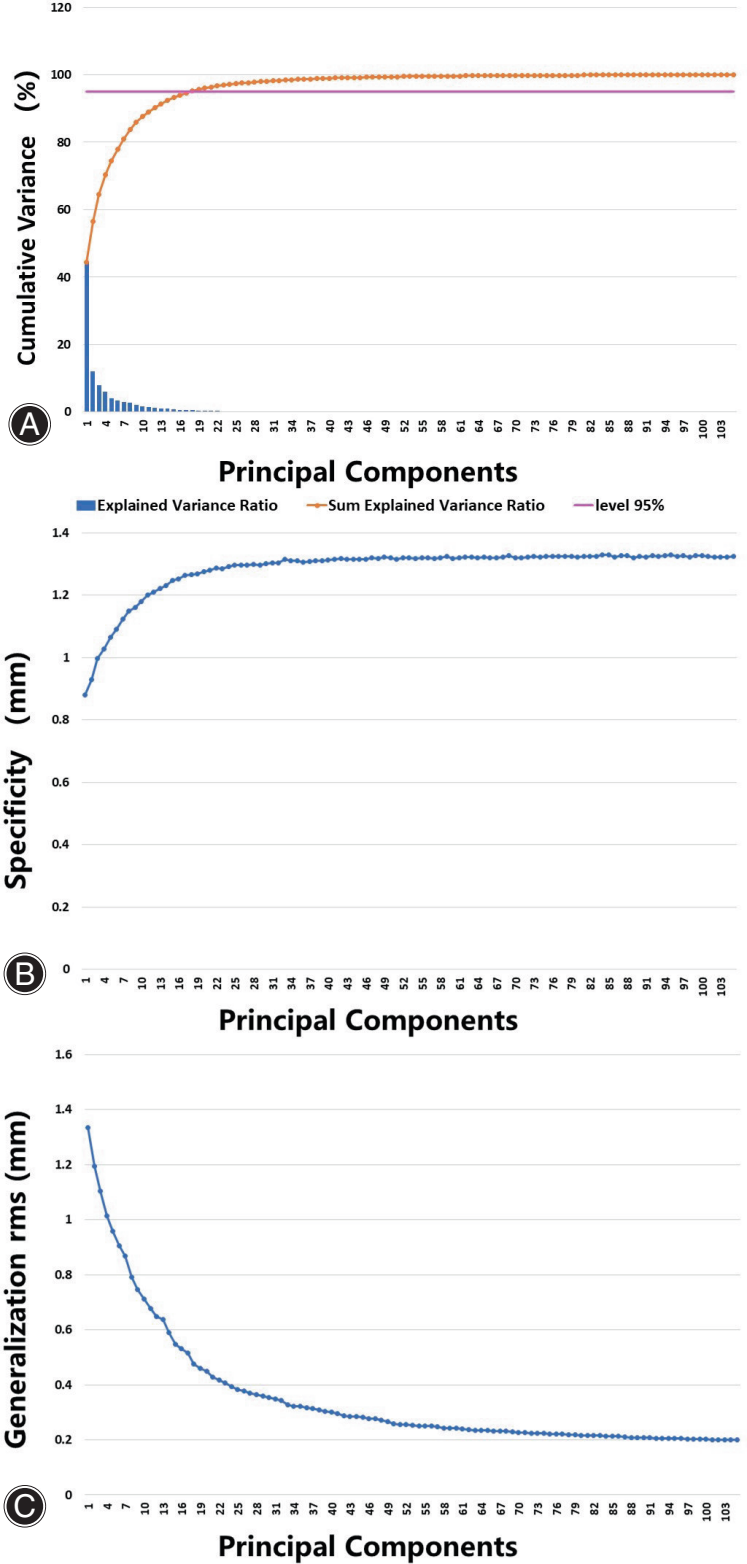
Evaluation of the SSM quality. (A) shows the compactness of the SSM; (B) shows the specificity of the SSM; (C) shows the generalization of the SSM.

Figure 5 shows the compactness, generalization and specificity of the SSM constructed from the 106 distal humeri. The compactness graph shows that the first 10 PCs explained approximately 87.6% of the variation in the training data set. To explain 95% of the variability, 18 PCs were needed. The generalization measurement shows that a random distal humerus can be reconstructed with an average error of 0.2 mm if all modes of variation are used. The specificity of the SSM ranged from 0.88 to 1.33 mm.

### Principal Components

The first seven PCs each represented more than 3% of the total variation (44.4%, 12.2%, 7.9%, 5.9%, 4.1%, 3.4% and 3%, respectively). These PCs jointly accounted for 80.9% of the variability. The measurements of the mean model and ± 3 SD models of the seven PCs are presented in Table 1. In the mean model, the distance between the medial and lateral epicondyles (Wtel) was 57.4 mm, the width of the articulation (Wcline) was 42.1 mm, and the angle of the TEL and C line (ATC) was 4.8°.

**Table 1:** Measurements of the mean mode and ± 3 SD modes of each variation

	Mean	PC1 + 3SD	PC1 – 3SD	PC2 + 3SD	PC2 – 3SD	PC3 + 3SD	PC3 – 3SD	PC4 + 3SD	PC4 – 3SD	PC5 + 3SD	PC5 – 3SD	PC6 + 3SD	PC6 – 3SD	PC7 + 3SD	PC7 – 3SD
Wtel (mm)	57.4	44.4	71.1	57.5	57.5	60.8	54.3	57.7	57.2	55.7	59.6	59.1	55.9	60.2	54.6
Wcline (mm)	42.1	32.9	52	42	42	44.3	40.4	41.8	42.2	41.6	43	41.2	43	43.8	40.9
Wcap (mm)	15.2	11.6	17.9	14.2	14.2	15.7	13	13.2	13.9	13.1	14.9	14.6	15.2	15.3	14
Wtro (mm)	19.2	12.9	20.7	17.6	17.6	18.3	16.1	16.7	17.6	16	18.6	16.8	17.1	18.5	16.1
RT	1.49	1.29	1.61	1.42	1.42	1.56	1.5	1.38	1.32	1.39	1.54	1.36	1.45	1.39	1.62
RTC	1.26	1.11	1.16	1.24	1.24	1.17	1.24	1.26	1.27	1.22	1.25	1.15	1.13	1.21	1.15
ATC (°)	4.8	4.6	6	5.6	5.6	5	5.3	6.7	3.9	6	3.9	4	6.8	4	6.9
Agro(°)	48.6	48.1	51.9	49.9	50.2	55.1	42.5	47.5	54.2	44.5	53.4	53.1	50	40.8	59.5
R1 (mm)	11.4	9.1	15	12	11.8	12.4	11.3	11.9	11.8	12.2	11.5	11.4	12.3	11.8	12
R2 (mm)	8.5	6.9	10	8.4	8.3	8.7	8.1	8.6	8.2	8.8	7.8	7.8	9	8.1	8.4
R3 (mm)	9.2	7.6	11	9.4	9.4	9.8	9	9.3	9.3	9.4	9.4	8.8	10	9.3	9.3
R4 (mm)	8.6	7.3	10.3	8.5	8.5	9.4	8.2	8.8	8.8	8.8	8.9	8.3	9.2	8.8	9.1
R5 (mm)	9.9	7.9	12.3	10	9.8	10.4	9.4	9.9	9.9	9.8	10.1	9.5	10.3	10.3	9.6
R6 (mm)	12.2	8.7	14.9	11.7	11.7	12.8	10.8	12.3	12.3	12.5	12.1	11.2	12	10.7	12.5

**Fig. 6:**
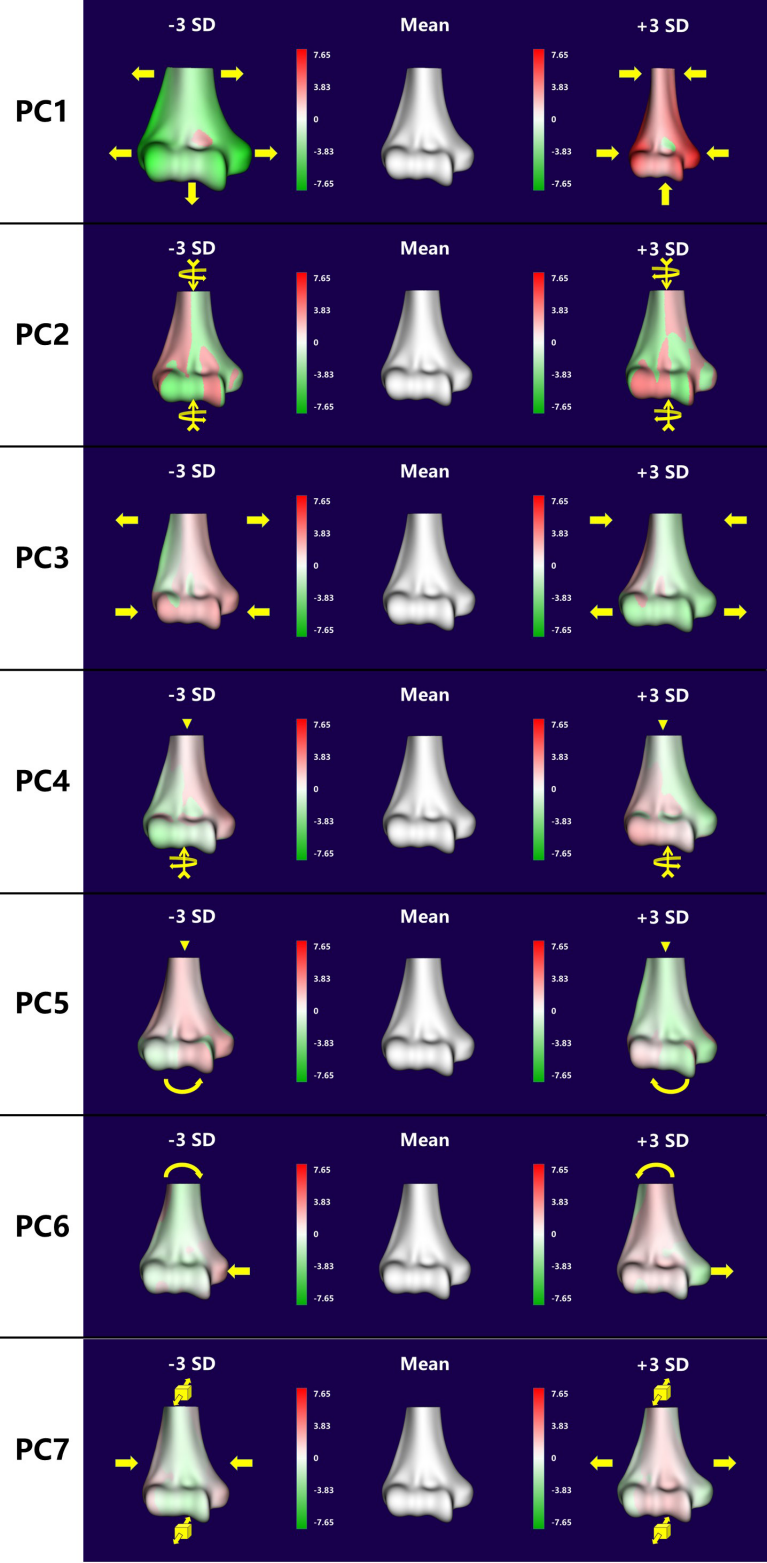
Seven variation modes of distal humerus. The arrows show the effect of each PC on the shape along the positive direction of that PC (Arrows with a vane at. show the directions of out‐of‐plane rotations).

Figure 6 shows the seven modes of shape variation in the distal humerus. The first PC represents the whole size variation of the distal humerus. Wtel varied from 44.4 to 71.1 mm, Wcline varied from 32.9 to 52 mm, and each component of articulation varied in the maximum range in size compared with those of other PCs. In the second PC, the whole distal humerus presented slight axial rotation (−3.7°–3.7°), while each measurement of the ±3 SD models did not show an apparent difference. In the third PC, the distal humerus presented a gradual variation of contraction or expansion, while the opposite was observed for the proximal area. Accordingly, the articulation size increased from the −3 SD to +3 SD model, including Wtel, Wcline, Wtro, Wcap and R1 to R6. In the fourth and fifth PCs, the articular component presented two patterns of slight rotation. The former represented with a rotation around the z‐axis (−2.5°–2.5°), and the latter around the y‐axis (−3.4°–3.4°). In addition, ATC and Agro varied over a large range. In the sixth PC, the most obvious changes were the size of the medial humeral epicondyle and the angle between the TEL and the shaft (94.7°–100.8°). The seventh PC presented a pattern of variation that contracted or expanded reversely between the width of the subcondylar area and the anteroposterior diameter of the supracondylar area, and Agro varied in the maximum range among the seven PCs (40.8°–59.5°).

### Evaluation of articular surface differences

**Fig. 7:**
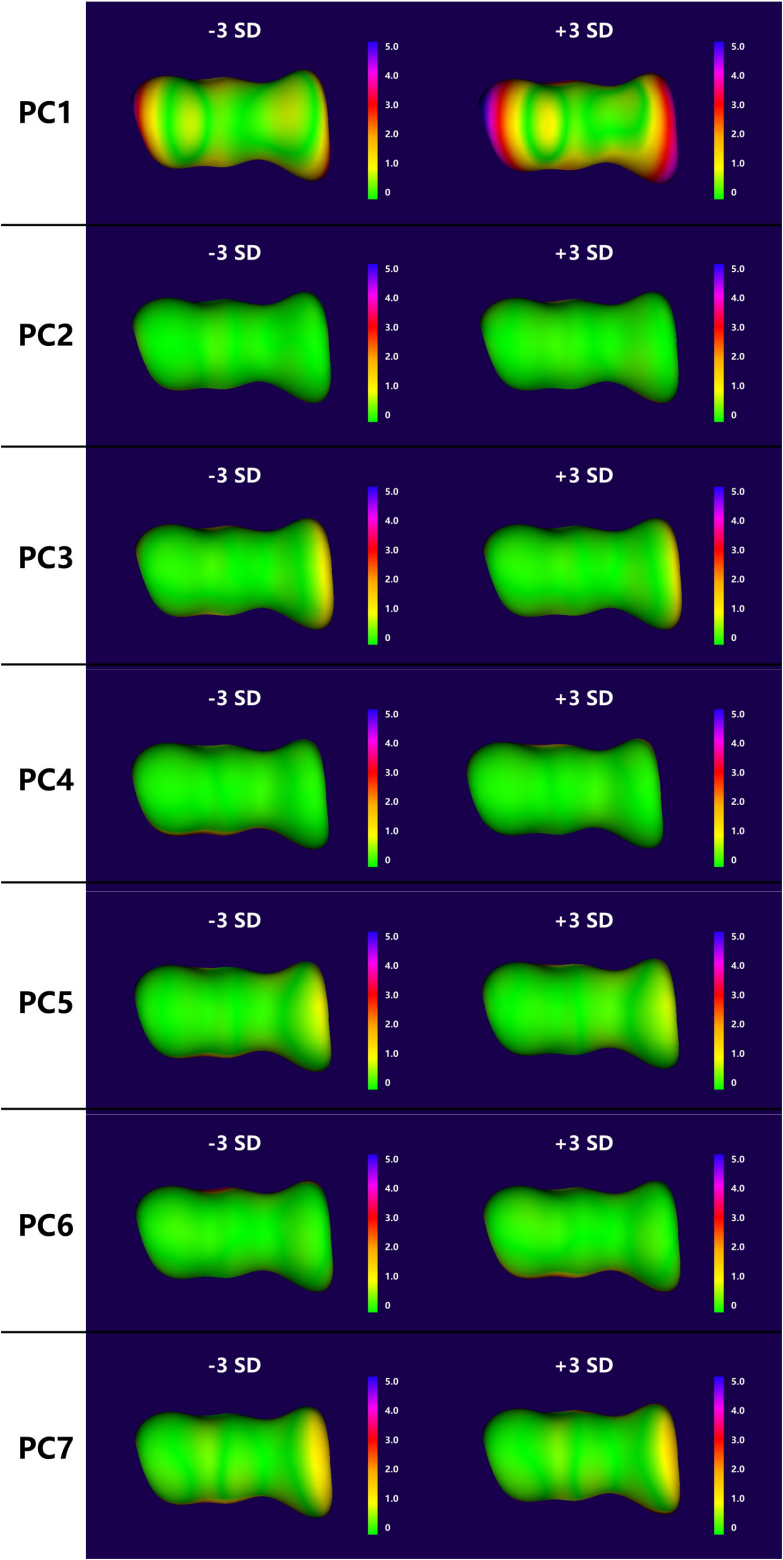
Articular surface differences between the mean model and ± 3 SD models for the seven PCs.

**Fig. 8:**
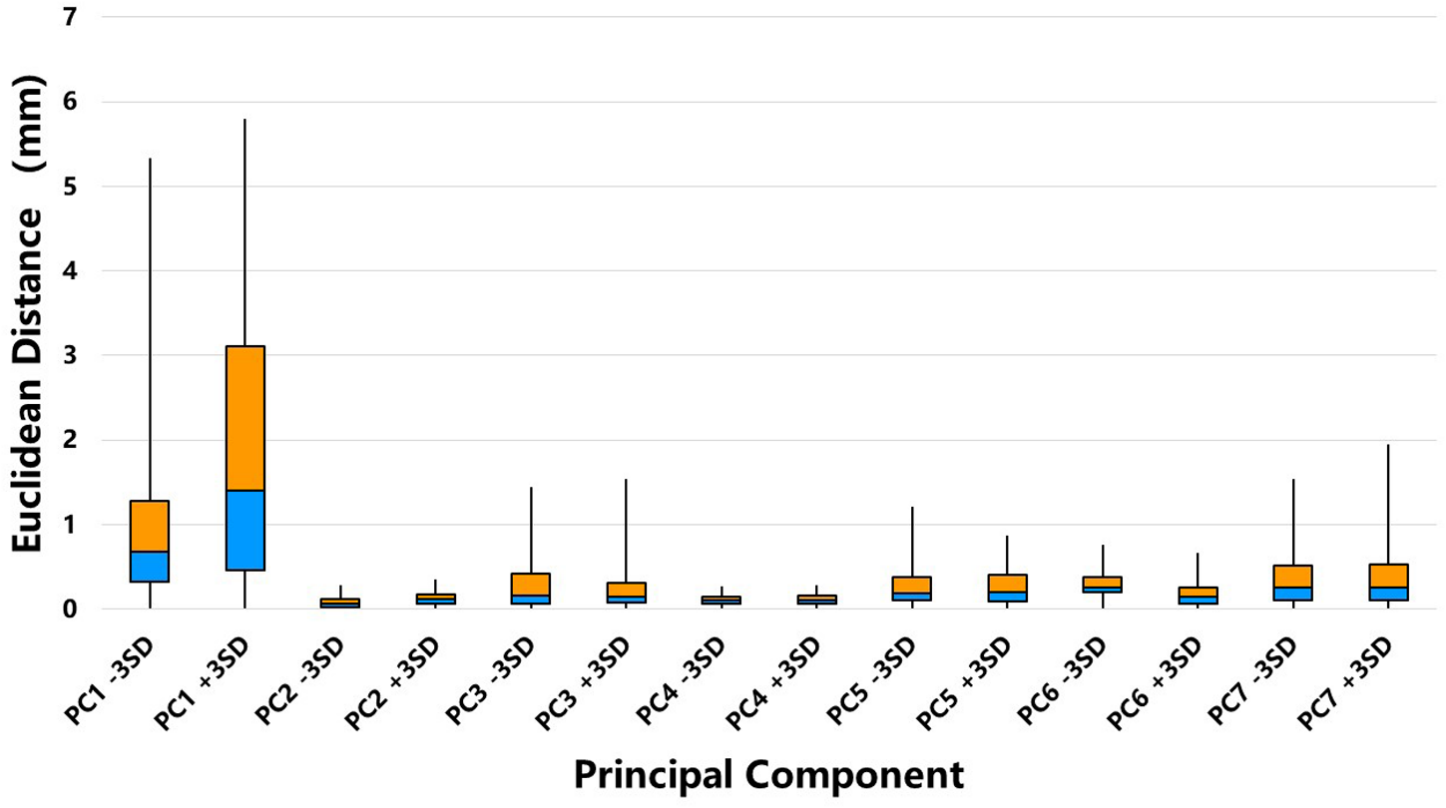
The boxplot of articular surface distances of the seven PCs.

Figure 7 shows the articular surface differences between the mean model and ± 3 SD models for the seven PCs. In the first PC, the articular surface difference was significant (RMS: 1.43 mm in the −3 SD model and 2.38 mm in the +3 SD model), with the maximum distance exceeding 5 mm. In some variations (PC3 ± 3 SD, PC5‐3 SD and PC7 ± 3 SD), the articular surface deformation was more than 1 mm to the maximum. Under other conditions, the differences were not remarkable despite the maximum deformation not exceeding 1 mm (Table 2, Fig. 8).

**TABLE 2:** The Euclidean point‐to‐mesh distance measured from all nodes of the mean articular mode to the ±3 SD articular surfaces

	Range (mm)	Median (mm)	Mean(mm)	Std Deviation (mm)	RMS (mm)
PC1 + 3SD	0 – 5.8	1.4	1.83	1.53	2.38
PC1 – 3SD	0 – 5.33	0.68	1.01	1.01	1.43
PC2 + 3SD	0 – 0.35	0.12	0.13	0.08	0.15
PC2 – 3SD	0 – 0.29	0.07	0.08	0.06	0.1
PC3 + 3SD	0 – 1.54	0.15	0.27	0.34	0.43
PC3 – SD	0 – 1.45	0.16	0.35	0.41	0.53
PC4 + 3SD	0 – 0.28	0.11	0.11	0.06	0.13
PC4 – 3SD	0 – 0.27	0.1	0.1	0.06	0.12
PC5 + 3SD	0 – 0.87	0.2	0.26	0.2	0.33
PC5 – 3SD	0 – 1.21	0.19	0.28	0.26	0.38
PC6 + 3SD	0 – 0.67	0.15	0.18	0.14	0.23
PC6 – 3SD	0 – 0.76	0.25	0.29	0.15	0.33
PC7 3SD	0 – 1.95	0.26	0.43	0.45	0.62
PC7 – 3SD	0 – 1.54	0.25	0.4	0.4	0.56

## Discussion

EHA is a rational technique for managing acute unreconstructible distal humeral fractures.^1^, ^2^, ^3^, ^4^, ^5^ Compared with TEA, EHA has multiple benefits, such as reducing postoperative restriction, preserving the bone stock and native collateral ligaments and shortening the operation time. Kwak *et al*.^2^ performed a meta‐analysis of EHA. They included nine studies with 115 patients and reported that cartilage wear was the most common complication (39.1% of the total), considerably higher than other complications, including heterotopic ossification (33%), implant loosening (11.3%), neuropathy (9.6%) and stiffness (5.2%). The reasons for cartilage degeneration in hemiarthroplasty are multifactorial. In regard to the distal humerus, which possesses relatively complicated articular components, the morphological error between the implant and the native articular surface is thought to be the primary reason. Therefore, it is necessary to perform a detailed analysis and thoroughly understand the morphology of distal humeral articulation. In this study, we evaluate the morphology of the distal humerus in healthy Chinese individuals and investigate the proper articular shape differences, which could help in the creation of anatomic prostheses, thus addressing the complications of EHA.

### Application of SSM in Orthopedics

Digital orthopedics was raised in China for many years, which is the application of computer science or digital techniques in clinical orthopedics facilitates the development of medicine.^25^ As one of important digital techniques, SSMs are widely used in the field of orthopedics, such as morphological analysis of human bony structures.^19^, ^21^, ^26^, ^27^, ^28^ It is capable of systematically describing the morphology and the main modes of 3D shape variation from highly variable and complex bony structures in terms of the pattern dimension reduction analysis called PCA. PCA can transform data from a high‐dimensional space to a space of fewer dimensions that can efficiently capture the main modes along which bony shape may vary.^29^ Soltanmohammadi *et al*.^27^ developed an SSM of the entire humerus using the CT scans of 75 humeri. However, the shape variation of the humerus was merely described in the proximal part. Vlachopoulos *et al*.^30^ constructed an SSM to predict the patient‐specific anatomy of the proximal or distal part of the humerus. However, they did not report the measurements for their models. To our knowledge, this study is the first to evaluate the morphology and variations of the distal humerus by means of an SSM.

### SSM of Distal Humerus

In our study, 106 samples of the humerus were clipped from the axial plane 50 mm proximal to the vertex of the olecranon fossa to eliminate the shape variation of other bony structures of the humerus, which would affect the result. Seven PCs formed the main modes of variation in the distal humerus (more than 80% variability). The first variation mode was the variation in size, accounting for 44.4%. The second PC represents a medial or lateral rotation of the distal humerus, which accounted for 12.2%. The third and seventh PCs revealed two patterns of variation in which the proximal and distal areas would contract or expand reversely between each other. The fourth and fifth PCs presented two patterns of slight rotation on the articulation component. In the sixth PC, the size of the medial humeral epicondyle varied with increasing PC score. As shown in Table 1, the distal humerus possesses complicated morphological variation modes. The table also shows that all PCs except the second one could have a certain impact on the shape of the articular surface.

### Geometric Measurements

In the current study, geometric measurements were performed on the variation models of the seven PCs. Table 3 shows the geometric data of the distal humerus according to previous studies published by orthopedic researchers. Hamasaki *et al*.^31^ was the first to thoroughly quantify the anatomical shape and size of the elbow. In his study, 36 Japanese cadaveric elbows were measured via microradiograms of sectioned samples and external contours. Fixing the axes of the humerus and ulna, they set the centerline between the medial and lateral epicondyles as a standardized line for elbow measurement. They performed measurements on both sides and reported a mean diameter of the central groove of 17.6 mm (right) and 17.7 mm (left) and a median trochlear flange of 24.5 mm (right) and 22.6 mm (left). This measurement of the Japanese skeleton is similar to the present study, which found the mean radii of the trochlear groove and median trochlear flange to be 8.5 and 11.4 mm, respectively. However, they did not report the proper width of the trochlea and alternatively measured the width at the margins along three directions.

**TABLE 3:** The geometric data of the distal humerus according to previous studies published by orthopedic researchers

Author (year)	Number	Origin	Methods	Trochlea				Capitellum			Width of TEL	Width of c line
				Width	Medial flange	Groove	Lateral flange	Width/height	Sagittal	Coronal		
Hamasaki *et al*. (1983) ^31^	36	Japan	Measure on sectioned samples	R: 21.4 ± 2.6 mm (AM); 23.5 ± 2.8 mm (DM); 23.9 ± 3.6 (PM) L: 21.1 ± 2.8 mm (AM); 23.3 ± 2.6 mm (DM); 23.5 ± 2.8 mm (PM)	NA	R: 17.6 mm (d); L: 17.7 mm (d)	R: 24.5 mm; L: 22.6 mm (d)	NA	NA	NA	NA	NA
Shiba *et al*. (1988)^17^	4	Canada	Measure on sectioned samples	NA	11.8–14.7 mm (r)	8.4–9.2 mm (r)	9.6 to 11.6 mm (r)	NA	9.6–12 mm (r)	NA	53.8– 69.1 mm	NA
Wevers *et al*. (1985)^16^	6	Canada	Measure on sectioned samples	21.3– 26.3 mm	24.5–30.3 mm (d)	14.9–18.4 mm (d)	NA	NA	19.2–23.7 mm (d)	NA	NA	40.0–49.4 mm
Sabo *et al*. (2011)^18^	50	Canada	3D reconstruction	NA	NA	NA	NA	13.9 ± 2.3 mm (W) 23.2 ± 2.8 mm (H)	11.6 ± 1.4 mm (r)	14.0 ± 3.0 mm	NA	NA
Desai *et al*. (2014)^14^	50	Canada	3D reconstruction	21.0 ± 2.6 mm	30.0 ± 4.1 mm (d)	17.9 ± 2.0 mm (r)	21.9 ± 2.3 mm (r)	17.2 ± 1.9 mm (W) 23.3 ± 2.3 (H)	9.0 ± 1.0 mm (r)	NA	NA	42.5 ± 4.6 mm

Abbreviations: AM, anterior margin; d, diameter; DM, distal margin; H, Height; L, left; NA, nonapplicable; PM, posterior margin; r, radius; R, right; TEL, transepicondylar line; W, width.

Shiba *et al*.^17^ and Wevers *et al*.^16^ performed similar measurements on a small sample of cadaveric samples. They sectioned the distal humeri in the sagittal plane and created circle fits for each slice to determine the center of each circle. The C line was referred to as the connecting line of each center. Shiba *et al*. reported the widths of TEL, sagittal radii of the capitellum, lateral trochlear flange, trochlear groove and medial trochlear flange of 53.8– 69.1, 9.6–12, 9.6–11.6, 8.4–9.0 and 11.8–14.7 mm, respectively.^17^ The trochlear and capitellar widths were not included in Shiba's study. In Wevers *et al*.'s study, the widths of the trochlea and capitellum ranged from 21.3 to 26.3 mm and from 15.5 to 19.1 mm, respectively. Wever *et al*. also measured the capitellar height (19.2–23.7 mm) and the diameters of the trochlear groove and the median trochlear flange (14.9–18.4 and 24.5–30.2 mm). Depending on the variation mode in the current study, the measurements mentioned above demonstrated different ranges (Table 1). The data of Shiba *et al*.'s and Wevers *et al*.'s studies are within the variance of our study, but the small sample size limits the ability to statistically compare them.

Desai *et al*.^14^ created three‐dimensional models from the CT scans of 50 human cadaveric distal humeri. After establishment of a coordinate system, circle fits were applied to slices sectioned along the articular flexion‐extension axis. The geometric centers of each slice were connected and fitted as the C line. They reported that the trochlear and capitellar widths were 21 ± 2.6‐ and 17.2 ± 1.9 mm, respectively. The sagittal diameters of the lateral trochlear flange, trochlear groove and medial trochlear flange were 21.9 ± 2.3, 17.9 ± 2 and 30 ± 4.1 mm, respectively. Sabo *et al*.^18^ performed morphologic analysis using a similar method but merely focused on the capitellum. Apart from the capitellar width and height, they found that the sagittal radius and the transverse radius were 11.6 ± 1.4 and 14 ± 3 mm, respectively. In the present study, the maximum size of the distal humerus appeared in PC1‐3SD (Table 1). These measurements are significantly smaller than Desai *et al*.'s and Sabo *et al*.'s data, which are based on Caucasian data.

### Articular Deformation Analysis

Furthermore, the proper articular deformation was calculated between the mean model and the maximum variation models in each PC. Landmark‐based registration was performed on the articular surface to reduce the position bias of articulation components. We found that in the first PC, the RMS was larger than 1 mm (the maximum distance was more than 5 mm). The trochlear and capitellar surfaces could both present significant deformations (Fig. 6). In PC3, PC5 and PC7, the articular surface could present with more than a 1 mm difference in the trochlear area. In other PCs, the differences were not remarkable even though the maximum deformation did not exceed 1 mm. Therefore, it could indicate that even though the distal humeral morphology possessed multiple complex variation modes, the proper shape of the articular surface primarily transformed in the first PC. Such data can provide a meaningful support for designing a universal prosthesis of EHA.

The endurable intra‐articular surface step‐off is not accurately defined in the treatment of intra‐articular fracture.^32^ In most studies, good clinical outcomes can be obtained with less than 2 mm displacement on the articular surface.^33^, ^34^ Limited step‐off and incongruity between articular surfaces could be accommodated given the viscoelastic properties possessed by the cartilage of native joints. Miyamura *et al*.^35^ created cartilage‐bone models using a laser scanner based on 20 elderly cadaveric elbows. They compared the models with noncartilage samples and calculated the thickness of the cartilage in each area. In the proximal part of the ulna, the thickness of the cartilage was more than 2 mm, and in the radial head, it was more than 1 mm. It can be inferred that the limited deformation of the trochlea and capitellum could be accommodated by the contralateral cartilage. To test and verify this speculation, further experiments are needed.

In a study by Willing *et al*.,^11^ reverse‐engineered EHA prostheses were manufactured and tested on cadaveric elbows. By simulating the motion of the elbow, they compared the articular mechanics between the native joints and subject‐specific prostheses. They found that reverse‐engineered prostheses did not reproduce the same contact pattern as the native joints. The possible reason was neglect of the thickness of the cartilage layer and the high stiffness of the metallic implants. In addition, they performed a finite element modeling study and found that the reverse‐engineered prostheses provided small improvements in contact mechanics compared with commercially available implants.^10^ They further designed a shape optimization technique by increasing the size of the implants.^13^ Significant improvements to minimize peak contact stresses were reported. Beyond shape optimization, the use of lower stiffness materials was also attempted to improve hemiarthroplasty contact mechanics.^12^ Therefore, the optimization in both morphology and materials could enable EHA to be a clinically reliable treatment for complex fractures of the distal humerus.

### Strengths and Limitations

In this study, we used a novel method (SSM) to evaluate the 3D morphology of the distal humerus in healthy Chinese individuals and to investigate the proper articular shape differences. However, this study has several limitations. First, larger sample size is needed to further verify and calculate the optimal data for developing EHA in Chinese population. Also, we did not analyze the geometric differences between the bilateral and bisexual groups. Finally, to provide more valid suggestions on the prosthesis design, more experimental studies are needed to investigate the speculation generated by this study on the joint mechanics of the elbow.

### Conclusion

In conclusion, a novel method (SSM) is described, which accurately evaluates the osseous anatomy and the shape variation of the distal humerus. The Chinese database produced here reflects the population‐specific characteristics of the bony structures. In our study, although the distal humeral morphology possessed complex variation modes, the proper shape of the articular surface primarily transformed into one particular variation pattern that was relevant to the bone size. This new information can be useful in the development of EHA implants in the future.

## Author Contributions

Wei Zhao, Yao Guo: Conceptualization, Methodology, Software, Data curation, Writing‐Original draft preparation. Chuangye Xu, Shiva Basnet, Yanjun Pei: 3D modeling, Validation, Writing‐Original draft preparation. Guoxian Pei, Xiuyun Su: Conceptualization, Methodology, Supervision, Writing‐Reviewing and Editing.

## Ethics Statement

This study involving human participants was reviewed and approved by the ethics committee of the Southern University of Science and Technology Hospital (No. of IRB: ECSUSTH‐2022‐064). The patients provided their written informed consent to participate in this study.

## Funding Information

This study was supported by the Research Startup Fund of the Southern University of Science and Technology (Y01416214).
